# The genetic diversity within the 1.4 kb *HLA-G* 5′ upstream regulatory region moderately impacts on cellular microenvironment responses

**DOI:** 10.1038/s41598-018-24009-7

**Published:** 2018-04-04

**Authors:** Fabrício C. Dias, Bruna C. Bertol, Isabelle Poras, Bruno M. Souto, Celso T. Mendes-Junior, Erick C. Castelli, Laure Gineau, Audrey Sabbagh, Nathalie Rouas-Freiss, Edgardo D. Carosella, Eduardo A. Donadi, Philippe Moreau

**Affiliations:** 1 Department of Medicine, Division of Clinical Immunology, Faculty of Medicine of Ribeirão Preto, University of São Paulo, Ribeirão Preto, ZIP Code 14.049-900 Brazil; 2 Commissariat à l’Energie Atomique et aux Energies Alternatives, Direction de la Recherche Fondamentale, Institut de Biologie François Jacob, Service de Recherches en Hémato-Immunologie, Hôpital Saint-Louis, Paris, ZIP code 75010 France; 3 Université Paris-Diderot, Sorbonne Paris-Cité, UMR_E5, Institut Universitaire d’Hématologie, Hôpital Saint-Louis, Paris, ZIP code 75010 France; 4 Department of Biochemistry and Immunology, Faculty of Medicine of Ribeirão Preto, University of São Paulo, Ribeirão Preto, ZIP Code 14.049-900 Brazil; 5 Departamento de Química, Faculdade de Filosofia, Ciências e Letras de Ribeirão Preto, Universidade de São Paulo, Ribeirão Preto, ZIP Code 14.049-900 Brazil; 6 São Paulo State University (UNESP), Department of Pathology, School of Medicine, Botucatu, State of São, Paulo, ZIP Code 18.618-687 Brazil; 7 UMR 216-MERIT, Institut de Recherche pour le Développement, Faculté de Pharmacie de Paris - Université Paris Descartes, COMUE Sorbonne Paris-Cité, Paris, ZIP Code 75006 France

## Abstract

The *HLA-G* 5’URR extending 1.4 kb from the ATG presents a unique set of regulatory elements among *HLA* genes. Several variable sites have been described that coincide with or are close to these elements, thus *HLA-G* 5′URR polymorphism might influence the *HLA-G* expression level. We cloned the ten most frequent *HLA-G* 5′URR haplotypes to evaluate their activity on a luciferase reporter gene in HLA-G^+^ cell lines (JEG-3/choriocarcinoma and FON^+^/melanoma). We also investigated associations between the plasma HLA-G (sHLA-G) levels and the *HLA-G* 5′URR variability in 157 healthy individuals. Cell lines were transfected with pGL3-Basic vector constructions containing *HLA-G* 5′URR sequences. The G010101a (in JEG-3) and G010101b (in FON^+^) haplotypes exhibited higher promoter activity, whereas the G010101d (in JEG-3) and G010102a (in FON^+^) haplotypes exhibited lower promoter activity. In the presence of *HLA-G* inducers (interferon-β and progesterone) or repressors (cyclopamine) *HLA-G* promoter activity was modulated, but certain haplotypes exhibited differential responses. No strict association was observed between plasma sHLA-G levels and the 5′URR haplotypes or genotypes; however, the G010101b haplotype was underrepresented among HLA-G-negative plasmas. Therefore, the *HLA-G* 5′URR polymorphism may have an impact on the modulation of *HLA-G* gene expression, but alone provides a limited predictive value for sHLA-G levels *in vivo*.

## Introduction

HLA-G is an immune checkpoint molecule that inhibits the function of immunocompetent cells such as the cytotoxic activity of NK and T CD8^+^ lymphocytes, through the binding to ILT-2/LIRB1, ILT-4/LIRB2 inhibitory receptors^1^. HLA-G is primarily expressed in cytotrophoblast cells and also in thymus, pancreas, cornea, nail matrix, mesenchymal stem cells, erythroid and endothelial precursors^2–5^. In pathological conditions, HLA-G may be neoexpressed in allograft tissues^6^ and in autoimmune disorders^7^, and its presence in these conditions has been associated with decreased morbidity and mortality. In contrast, the expression of HLA-G on tumour cells or chronically virus-infected cells may increase the morbidity of these disorders^8–12^. Interestingly, there are interindividual differences in the level of HLA-G expression in normal and pathological conditions suggesting that *HLA-G* gene polymorphisms, particularly variable sites located along the regulatory regions, have an impact on it.

The *HLA-G* gene presents limited coding region polymorphisms compared with classical HLA-class I genes, showing 58 alleles (IPD-IMGT/HLA, v3.31.0) that encode 20 different proteins, due to the presence of several synonymous mutations^13^. Nonetheless, many variable sites have been reported at *HLA-G* regulatory segments including the 3′untranslated and 5′upstream regions (3′UTR and 5′URR), and this variability has been hypothesized to be important for *HLA-G* expression. The 3′UTR presents at least 18 single nucleotide variations and a 14 base pair insertion/deletion motif^14–20^. These variations may affect microRNA binding and *HLA-G* mRNA stability^21,22^. In addition, we recently reported that 3′UTR haplotypes are associated with differential plasma soluble HLA-G (sHLA-G) levels in healthy individuals^23^, and differentially respond to endogenous cell factors^19^. On the other hand, the 5′URR here considered as the 1.4 kb upstream the first translated ATG, shows at least 35 SNPs defining 68 haplotypes, of which 9–10 have been frequently observed in worldwide populations^20,24–27^. With the exception of one prior study analysing five 5′URR haplotypes^28^, little is known about the impact of 5′URR variations on the *HLA-G* activity. *In silico* analyses revealed that 5′URR haplotypes can be clustered into four groups, shaped by balancing selection, which may characterize different promoter region activity^29,30^, likely due to putative differential transcription factor binding to regulatory elements.

When compared to classical *HLA-class I* promoters, several regulatory sequences are disrupted at the *HLA-G* proximal promoter region (enhancer-A, ISRE, X2 and Y sequences), except the S domain, a potential binding site to the RFX complex, and the X1 domain, a binding site to RFX in classical HLA-class I and HLA-class II promoters^31,32^. Several specific regulatory elements have been described within the 1.4 kb upstream segment, considered from the ATG (nucleotide + 1), that modulate *HLA-G* expression. Among them, the following are inducers: (i) a response element to progesterone (PRE) (−52 bp to −38 bp)^33^; (ii) a heat shock element (HSE) sequence (−464 bp to −453 bp), which presents a binding domain to heat shock transcription factor 1 (HSF1) in stress response^34^; (iii) an interferon-stimulated response element (ISRE) sequence (−745 bp to −754 bp), presenting an interaction domain with interferon regulatory factor 1 (IRF1) in response to interferon-β^35^; (iv) the cyclic AMP-response element (CRE) and TPA response element (TRE) sequence (−1387 bp to −1371 bp) are binding sites to cyclic AMP responsive element binding protein 1 (CREB1) and to the activating transcription factor 1 (ATF1)^36^. CREB1 also binds to two other sites (−941 bp to −935 bp and −777 bp to −771 bp)^36^. Other elements are repressors of the *HLA-G* expression: (i) the Ras responsive element binding protein 1 (RREB-1) that acts through three Ras response elements (RRE) (−1378 bp to −1358 bp, −157 bp to −143 bp, and −59 bp to −54 bp)^37^; and (ii) the glioma-associated oncogene-3 (*GLI3*) (−1116 bp to −1108 bp), a signal transducer of the Hedgehog pathway (HH) that is induced by cyclopamine treatment. *GLI3* is associated with decreased intracytoplasmic HLA-G5 level during osteoblast maturation^38^. Since many polymorphic sites were described surrounding these regulatory sites, they may influence regulatory protein binding and, thus, *HLA-G* transcription levels.

Considering that: (i) The *HLA-G* 5′URR presents peculiar response to transcription factors that may differ from classical HLA class I genes; (ii) several variable sites have been described at the *HLA-G* promoter segment, some of them coincide with or are close to transcription factor binding sites; (iii) little is known about the influence of *HLA-G* 5′URR variability on gene expression^24^, in this study we: (i) cloned the ten most frequent *HLA-G* 5′URR haplotypes observed worldwide and analysed their activity on a luciferase reporter gene in two HLA-G positive cell lines (choriocarcinoma JEG-3 and melanoma FON^+^) in the presence or absence of modulators (inducers: interferon-β and progesterone; repressor: cyclopamine) of the *HLA-G* expression, and (ii) determined *HLA-G* 5′URR alleles, genotypes, haplotypes, diplotypes and haplotype groups in healthy individuals to associate them with their plasma sHLA-G levels.

## Results

### The *HLA-G* 5′URR activity in HLA-G positive cells varies according to the cloned haplotype and to the cell type

We used two types of HLA-G positive cells to investigate the role of cell microenvironment on the *HLA-G* expression pattern according to 5′URR haplotype. Choriocarcinoma JEG-3 and melanoma FON^+^ cell lines were transfected, as previously described^19^, with pGL3-Basic vector constructions containing one of the ten most frequent *HLA-G* 5′URR haplotypes known as G0104a; G0104b; G010102a; G010101a; G010101b; G010101c; G010101d; G010101f; G0103a and G0103e.

**Figure 1:**
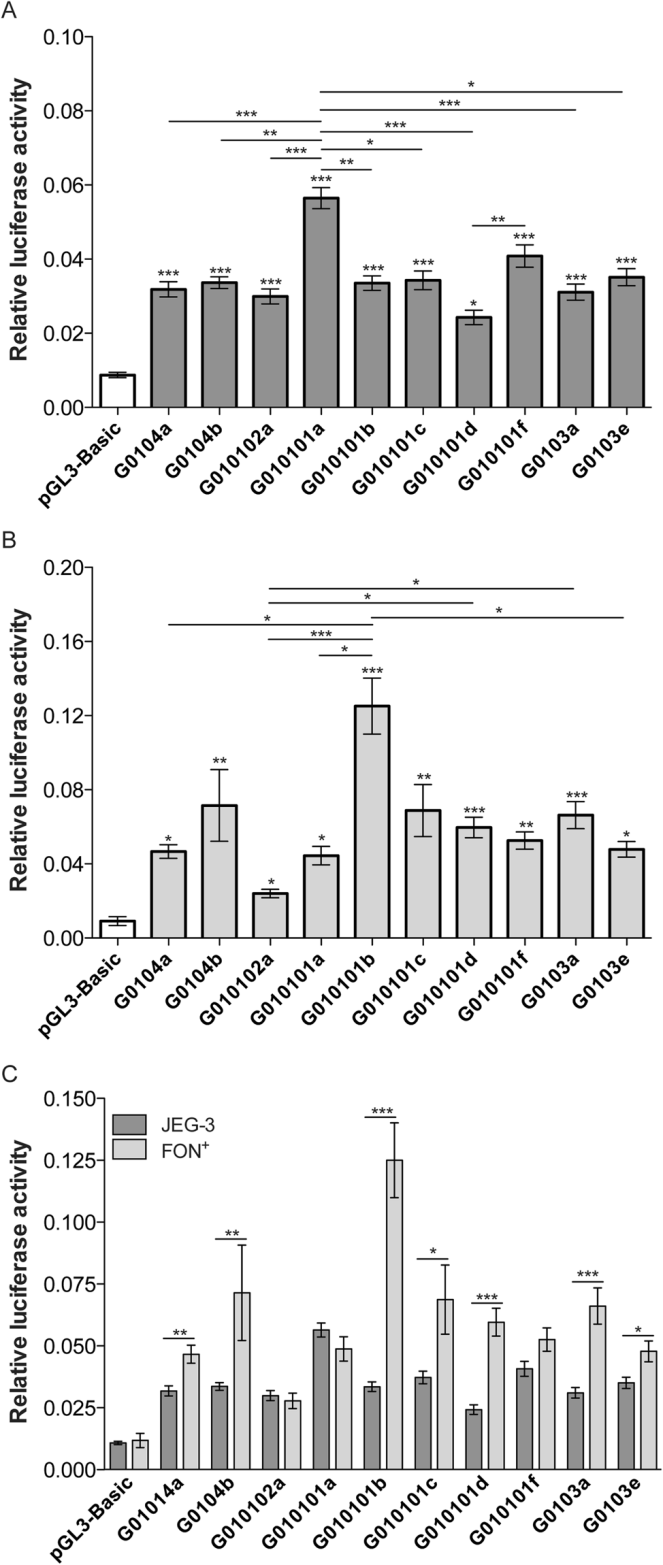
Luciferase activity from ten haplotypes of the *HLA-G* 5′URR in choriocarcinoma JEG-3 (**A**) and melanoma FON^+^ (**B**) cell lines. (**C**) Comparison between JEG-3 and FON^+^ cells of each transfected haplotype. The data represent the mean SEM. The twelve and five independent experiments were performed in duplicate for JEG-3 and FON^+^ cells, respectively. The Kruskal-Wallis test followed by the Dunn′s posttest was performed for A and B and the Mann-Whitney test was performed for C. *P < 0.05; **P < 0.01; ***P < 0.001.

Overall, we observed significant differences in luciferase expression levels among haplotypes for both JEG-3 (Fig. 1A) and FON^+^ (Fig. 1B) cells. Compared to cells transfected with an empty vector (luciferase activity background), all *HLA-G* 5′URRs transfected in JEG-3 cells induced the luciferase activity (*P* < 0.001 for all comparisons, except for the G010101d haplotype that showed *P* < 0.05). Compared with the empty vector, the G010101a haplotype was associated with the highest luciferase activity (6.5 fold) and the G010101d haplotype was the lowest one (2.8 fold) (Fig. 1A). Regarding FON^+^ cells, the G010101b and the G010102a haplotypes exhibited the highest (10.6 fold) and the lowest (2.4 fold) luciferase activity, respectively; however, high significance was reached for G0104b (*P* < 0.01), G010101b (*P* < 0.001), G010101c (*P* < 0.01), G010101d (*P* < 0.001), G010101f (*P* < 0.01) and G0103a (*P* < 0.001) haplotypes compared to the empty vector (Fig. 1B).

The highest luciferase activity observed for JEG-3 harbouring the G010101a haplotype was significantly different when compared to G0104a (*P* < 0.001), G0104b (*P* < 0.01), G010102a (*P* < 0.001), G010101b (*P* < 0.01), G010101d (*P* < 0.001), G0103a (*P* < 0.001) and G0103e (*P* < 0.05) haplotypes, while the lowest activity observed for G010101d haplotype was significantly different when compared only to G010101a (P < 0.001) and G010101f (P < 0.01) haplotypes (Fig. 1A). The highest luciferase activity observed for FON^+^ harbouring the G010101b haplotype was significant when compared to the ones observed for the G0104a (*P* < 0.05), G010102a (*P* < 0.001), G010101a (*P* < 0.05) and G0103e (*P* < 0.05) haplotypes, while the lowest activity for the G010102a haplotype was significantly different when compared to G010101b (*P* < 0.001), G010101d (*P* < 0.05) and G0103a (*P* < 0.05) haplotypes (Fig. 1B).

Since the relative luciferase activity observed in JEG-3 and FON^+^ cells was analysed using similar conditions, we compared the cloned 5′URR segments between these two cell types. Overall, the luciferase activity observed for FON^+^ cells was higher than the one observed for JEG-3 cells, except for the G010102a, G010101a and G010101f haplotypes. Noteworthy, the G010101b, G010101d and G0103a haplotypes exhibited the greatest differences (*P* < 0.0001 for all comparisons) (Fig. 1C).

### Differential activity of *HLA-G* 5′URR haplotypes in transfected JEG-3 cells exposed to activating (interferon-β and progesterone) or repressing (cyclopamine) agents

To further explore the 5′URR haplotype response to agents known to modulate *HLA-G* expression levels, JEG-3 cells transfected with each of the ten haplotypes cloned into pGL3-Basic vector were treated with 1000 U/mL interferon-β^28^, 1 µg/mL progesterone^39^ or 5 µM cyclopamine^38^. We performed individual comparisons of luciferase activity: i) before and after treatment for each construction (ratio or delta values), ii) comparisons among all haplotypes, and iii) comparisons according to the variable site close to the target site of the modulating agent.

**Figure 2:**
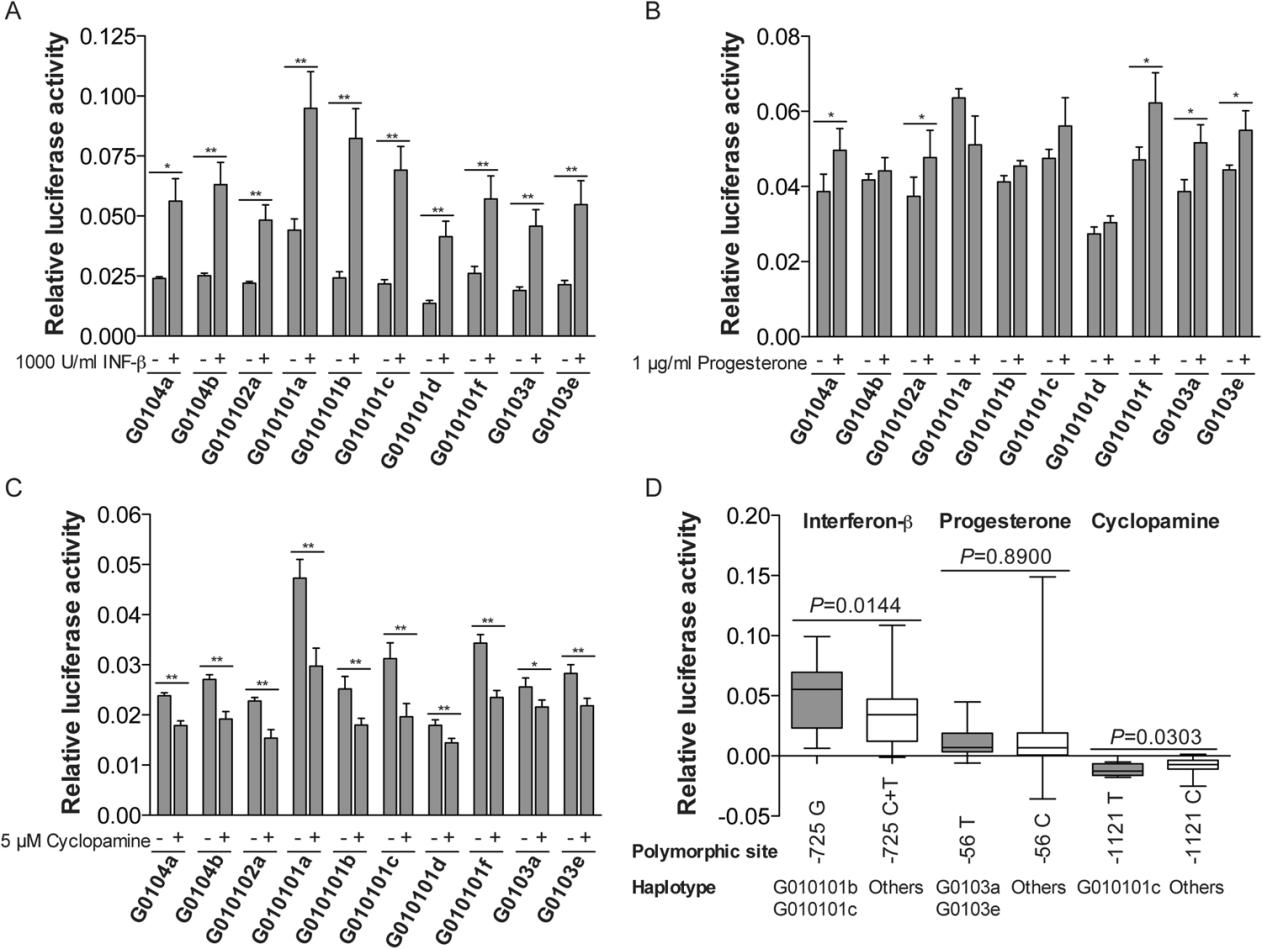
Luciferase activity from ten haplotypes of the *HLA-G* 5′URR in choriocarcinoma JEG-3 cell line treated with (**A**) 5 µM cyclopamine; (**B**) 1000 U/mL interferon-β; and (**C**) 1 µg/mL progesterone. The data represent the mean SEM. The Wilcoxon matched pairs test was used for comparisons between treated and untreated transfectants. *P < 0.05; **P < 0.01. (**D**) Association analysis of the specific polymorphic sites near to targets of IFN-β, progesterone and cyclopamine. The data represent the median with the minimum and maximum values. The statistical analysis was performed by Mann-Whitney test. The four independent experiments were performed in duplicate.

As expected, interferon-β treatment induced luciferase activity in cells transfected with all haplotypes when compared to non-transfected cells: G0104a (2.4-fold, *P* < 0.01), G0104b (2.5-fold; *P* < 0.05), G010102a (2.2-fold; *P* < 0.01), G010101a (2.2-fold; *P* < 0.01), G010101b (3.4-fold; *P* < 0.01), G010101c (3.5-fold; *P* < 0.01), G010101d (3.0-fold; *P* < 0.01), G010101f (2.2-fold; *P* < 0.01), G0103a (2.4-fold; *P* < 0.01) and G0103e (2.5-fold; *P* < 0.01). Noteworthy, the mean luciferase activity for the G010101b and G010101c transfectants increased (*P* < 0.01, for each comparison) more than three folds when compared to non-treated transfectants (Fig. 2A; Table 1). Notwithstanding, no significant difference was observed when the modulation of interferon-β treatment was compared across all ten haplotypes (ratio and delta values) (Table 1). Considering that (i) a functional *HLA-G* ISRE which responds to interferon β is close to the triallelic SNP at position −725 (alleles −725G, −725C and −725T) and (ii) G010101b and G010101c are the only haplotypes that present a Guanine at this position; these two haplotypes were grouped together and their activity levels after interferon-β treatment were compared to all other haplotypes exhibiting Cytosine or Thymine. Significant results were observed comparing either the ratio between post-treatment/pre-treatment expression (*P* = 0.02) or the difference (delta value) between post-treatment/pre-treatment (*P* = 0.01) (Fig. 2D). Therefore, haplotypes exhibiting a Guanine at the −725 position exhibited higher luciferase activity than the ones harboring Cytosine or Thymine at this position.

**Table 1:** Effect of the treatment with interferon-β, progesterone or cyclopamine of each *HLA-G* 5′URR constructions transfected into JEG-3 cell line.

Haplotypes	Treatment						
	Interferon-β		Progesterone		Cyclopamine		
	Delta	Ratio	Delta	Ratio	Delta	Ratio	1-Ratio
G0104a	0.0244	2.0990	0.0136	1.2240	−0.0056*	0.7618	0.2382
	0.0321	2.3500	0.0106	1.2330	−0.0060	0.7514	0.2486
	(0.0266)	(1.0770)	(0.0076)	(0.2010)	(0.0030)	(0.1186)	(0.1186)
	*n* = 8	*n* = 8	*n* = 8	*n* = 8	*n* = 10	*n* = 10	*n* = 10
G0104b	0.0414	2.6980	0.0040	1.0880	−0.0072	0.7462	0.2538
	0.0379	2.4490	0.0024	1.0580	−0.0079	0.7043	0.2957
	(0.0239)	(0.8426)	(0.0086)	(0.2097)	(0.0037)	(0.1551)	(0.1551)
	*n* = 8	*n* = 8	*n* = 8	*n* = 8	*n* = 10	*n* = 10	*n* = 10
G010102a	0.0240	2.0590	0.0082	1.1930	−0.0074	0.6861	0.3139
	0.0263	2.1520	0.0086	1.2140	−0.0074	0.6626	0.3374
	(0.0161)	(0.6401)	(0.0220)	(0.2048)	(0.0038)	(0.1862)	(0.1862)
	*n* = 8	*n* = 8	*n* = 8	*n* = 8	*n* = 10	*n* = 10	*n* = 10
G010101a	0.0428	2.1930	0.0020	1.0080	−0.0145*	0.5833*	0.4167*
	0.0507	2.1660	0.0337	1.2550	−0.0176	0.6172	0.3828
	(0.0361)	(0.7719)	(0.0595)	(0.5265)	(0.0055)	(0.1110)	(0.1110)
	*n* = 8	*n* = 8	*n* = 8	*n* = 8	*n* = 10	*n* = 10	*n* = 10
G010101b	0.0604	3.5240	0.0057	1.1540	−0.0065*	0.7248	0.2752
	0.0581	3.4360	0.0042	1.1120	−0.0072	0.7330	0.2670
	(0.0317)	(1.3240)	(0.0052)	(0.1234)	(0.0047)	(0.1247)	(0.1247)
	*n* = 8	*n* = 8	*n* = 8	*n* = 8	*n* = 10	*n* = 10	*n* = 10
G010101c	0.0488	2.9900	0.0027	1.0650	−0.0126^#^	0.6854	0.3146
	0.0474	3.5290	0.0086	1.1550	−0.0116	0.6180	0.3820
	(0.0307)	(2.1220)	(0.0156)	(0.2824)	(0.0049)	(0.1828)	(0.1828)
	*n* = 8	*n* = 8	*n* = 8	*n* = 8	*n* = 10	*n* = 10	*n* = 10
G010101d	0.0252	2.8540	0.0160	1.1810	−0.0032*^#^	0.7867	0.2133
	0.0278	2.9690	0.0181	1.1290	−0.0035	0.8063	0.1937
	(0.0160)	(0.8591)	(0.0194)	(0.1484)	(0.0020)	(0.0914)	(0.0914)
	*n* = 8	*n* = 8	*n* = 8	*n* = 8	*n* = 10	*n* = 10	*n* = 10
G010101f	0.0291	2.2470	0.0218	1.2920	−0.0119^#^	0.6982	0.3018
	0.0311	2.1530	0.0276	1.2940	−0.0109	0.6870	0.3130
	(0.0217)	(0.6191)	(0.0252)	(0.1730)	(0.0042)	(0.1137)	(0.1137)
	*n* = 8	*n* = 8	*n* = 8	*n* = 8	*n* = 10	*n* = 10	*n* = 10
G0103a	0.0263	2.5260	0.0063	1.1710	−0.0050*	0.8310*	0.1690*
	0.0267	2.3630	0.0130	1.4400	−0.0040	0.8574	0.1426
	(0.0170)	(0.7231)	(0.0174)	(0.6873)	(0.0041)	(0.1566)	(0.1566)
	*n* = 8	*n* = 8	*n* = 8	*n* = 8	*n* = 10	*n* = 10	*n* = 10
G0103e	0.0283	2.4090	0.0070	1.1590	−0.0083*	0.7512	0.2488
	0.0333	2.4690	0.0106	1.2260	−0.0064	0.7769	0.2231
	(0.0246)	(0.8758)	(0.0120)	(0.2440)	(0.0039)	(0.1336)	(0.1336)
	*n* = 8	*n* = 8	*n* = 8	*n* = 8	*n* = 10	*n* = 10	*n* = 10

Median, Mean expression, Standard Deviation (SD) and *n* = Sample sizes are represented. Delta = luciferase activity difference between treated and non-treated transfectants measured in the same experiment. Ratio = relative luciferase activity for treated and non-treated transfectants measured in the same experiment. 1-Ratio = 1 minus relative luciferase activity for treated and non-treated transfectants measured in the same experiment. The statistical analysis was performed using Kruskal-Wallis test followed by the Dunn’s posttest. *P < 0.05 indicating statistical significance compared to G010101a haplotype; ^#^*P* < 0.05 indicating statistical significance compared to G010101d haplotype.

The treatment of JEG-3 cells with progesterone provided significant increased luciferase activity only for five haplotypes; however, less intense than the one observed for interferon-β treatment. The treatment with progesterone increased mean luciferase activity in cells transfected with G0104a (1.2-fold higher; *P* < 0.05), G010102a (1.2-fold; *P* < 0.05), G010101f (1.3-fold; *P* < 0.05), G0103a (1.4-fold; *P* < 0.05) and G0103e (1.2-fold; *P* < 0.05) haplotypes (Fig. 2B). The comparisons of the progesterone effect across the ten haplotypes did not reveal significant differences (ratio and delta values) (Table 1). Considering that progesterone receptor binds to the −37 bp position at the *HLA-G* promoter region, and considering that a SNP at position −56 (−56C or −56T) is observed at the 5′URR, we evaluated the effect of this polymorphic site on luciferase activity induced by progesterone, and no significant differences were observed (*P* = 0.89) (Fig. 2D).

In contrast with interferon-β and progesterone, the treatment with cyclopamine decreased the mean luciferase activity in cells transfected with all haplotypes when compared to non-transfected cells: G0104a (−1.3-fold, *P* < 0.01), G0104b (−1.4-fold; *P* < 0.01), G010102a (−1.5-fold; *P* < 0.01), G010101a (−1.6-fold; *P* < 0.01), G010101b (−1.4-fold; *P* < 0.01), G010101c (−1.6-fold; *P* < 0.01), G010101d (−1.2-fold; *P* < 0.01), G010101f (−1.5-fold; *P* < 0.0001), G0103a (−1.2-fold; *P* < 0.05) and G0103e (−1.2-fold; *P* < 0.01). It may be noticed that the repressive effect was more pronounced for the G010101a and G010101c haplotypes (Fig. 2C). The comparison of delta values among haplotypes showed that G010101a exhibited a greater repression of the luciferase activity when compared to G0104a (*P* < 0.01); G010101b (*P* < 0.05), G010101d (*P* < 0.001), G0103a (*P* < 0.001), and G0103e (*P* < 0.05) haplotypes (Table 1). On the other hand, the G010101d haplotype exhibited the lesser effect of cyclopamine when compared to G010101c (*P* < 0.05) and G010101f (*P* < 0.05) haplotypes. The comparison of both ratio and 1-ratio values showed that the G010101a haplotype presented a higher repression by cyclopamine when compared to G0103a haplotype (*P* < 0.05) (Table 1). Considering that: (i) cyclopamine targets the *HLA-G* promoter region at positions −1116 to −1108 and (ii) there is a nearby polymorphic site at -1121 position (−1121T or −1121C), we evaluated the luciferase activity according to this polymorphism. Cyclopamine produced a more repressive effect on the haplotype containing the −1121T allele (G010101c) when compared to other haplotypes containing the −1121C allele (*P* = 0.03) (Fig. 2D).

### The *HLA-G* G010101b haplotypes may influence plasma sHLA-G levels

To investigate whether *HLA-G* 5′URR haplotypes can influence plasma sHLA-G levels, we evaluated the relationship between sHLA-G levels and the variability of the *HLA-G* 5′URR segment in 157 healthy Brazilian individuals.

**Figure 3:**
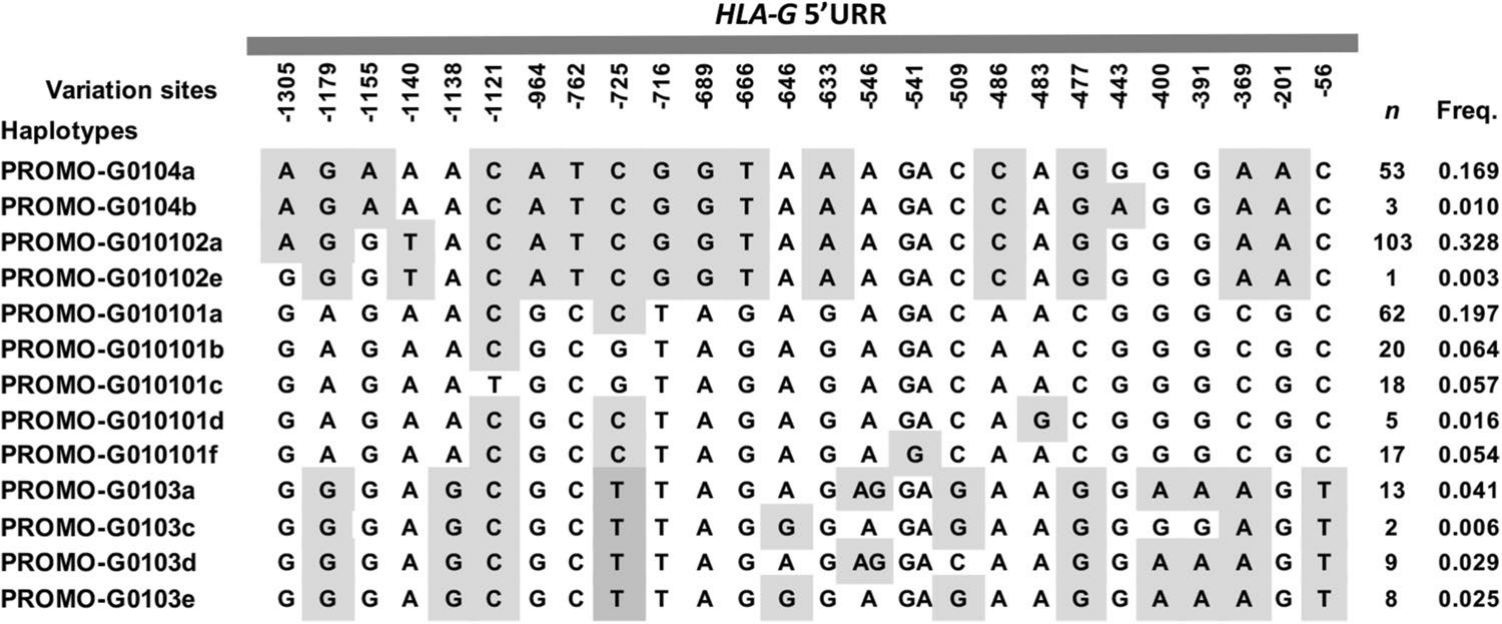
Variables sites at the *HLA-G* 5′URR and their haplotypes. The position of each variable site is determined considering the Adenine of the first translated ATG as +1. (^n^) number of individuals observed in each *HLA-G* 5′URR haplotype. (^Freq.^) Haplotype frequencies observed at the *HLA-G* 5′URR in this Brazilian population. The alternative alleles regarding the human genome draft version hg19 are marked in shades of gray.

We observed 26 variable sites at this segment, all of them previously described in the Brazilian population^20,30^. Genotype frequencies were in agreement with the Hardy-Weinberg equilibrium expectations and no new polymorphism was observed in this region. Thirteen different *HLA-G* 5′URR haplotypes were identified; all of them already described for Brazilians and for other population samples^20^. The most frequent haplotype was G010102a (32.8%) and the least frequent was the G010102e (0.3%) haplotype. Figure 3 shows all haplotypes identified in this study with their respective frequencies.

Soluble HLA-G levels in the whole sample ranged from 0.0 to 30.0 ng/mL (median = 1.8 ng/mL and mean = 4.6 ± 5.9 ng/mL). It should be emphasized that sHLA-G was not detected in 64 samples, a fact that results in a positive skew in sHLA-G level distribution and explain why the median is smaller than the mean. When the whole sample was stratified according to the presence (sHLA-G^+^; *n* = 93) or not (sHLA-G^−^; *n* = 64) of detectable sHLA-G, the median and mean values become quite similar in the sHLA-G^+^ group (7.4 ng/mL *vs*. 8.2 ng/mL).

The exact test of population differentiation based on haplotype frequencies revealed no difference between the sHLA-G^+^ and sHLA-G^−^ groups (*P* = 0.1010 ± 0.0104), but the G010101b haplotype was significantly underrepresented among sHLA-G^−^ when compared with sHLA-G^+^ plasmas (*P* = 0.047), indicating that this haplotype is related to a greater sHLA-G level (Table 2).

**Table 2:** Distribution of *HLA-G* 5′URR haplotypes in a Brazilian population sample stratified according to the presence or absence of HLA-G expression.

Haplotype	Absence of HLA-G expression (2*n* = 128)	Presence of HLA-G expression (2*n* = 186)	*P*-value (Fisher Exact Test)
**G0104a**	0.0391	0.0645	0.4484
**G0104b**	—	0.0161	0.2733
**G010102a**	0.0391	0.0806	0.1632
**G010101a**	0.3830	0.2960	0.1141
**G010101b**	0.1170	0.2040	**0.0469** ^*^
**G010101c**	0.0547	0.0161	0.0972
**G010101d**	0.0469	0.0161	0.1667
**G010101f**	0.0547	0.0591	1.0000
**G0103a**	0.2030	0.1940	0.8856
**G0103d**	0.0469	0.0376	0.7759
**G0103e**	0.0156	0.0161	1.0000

Exact test of differentiation based on haplotype frequencies does not reveal statistically significant differences between groups (0.1001 ± 0.0084). Statistically significant values are marked in boldface.

^*^This value is not significant at 5% level after the Bonferroni correction for multiple tests (corrected α = 0.05/11 = 0.0045).

**Figure 4:**
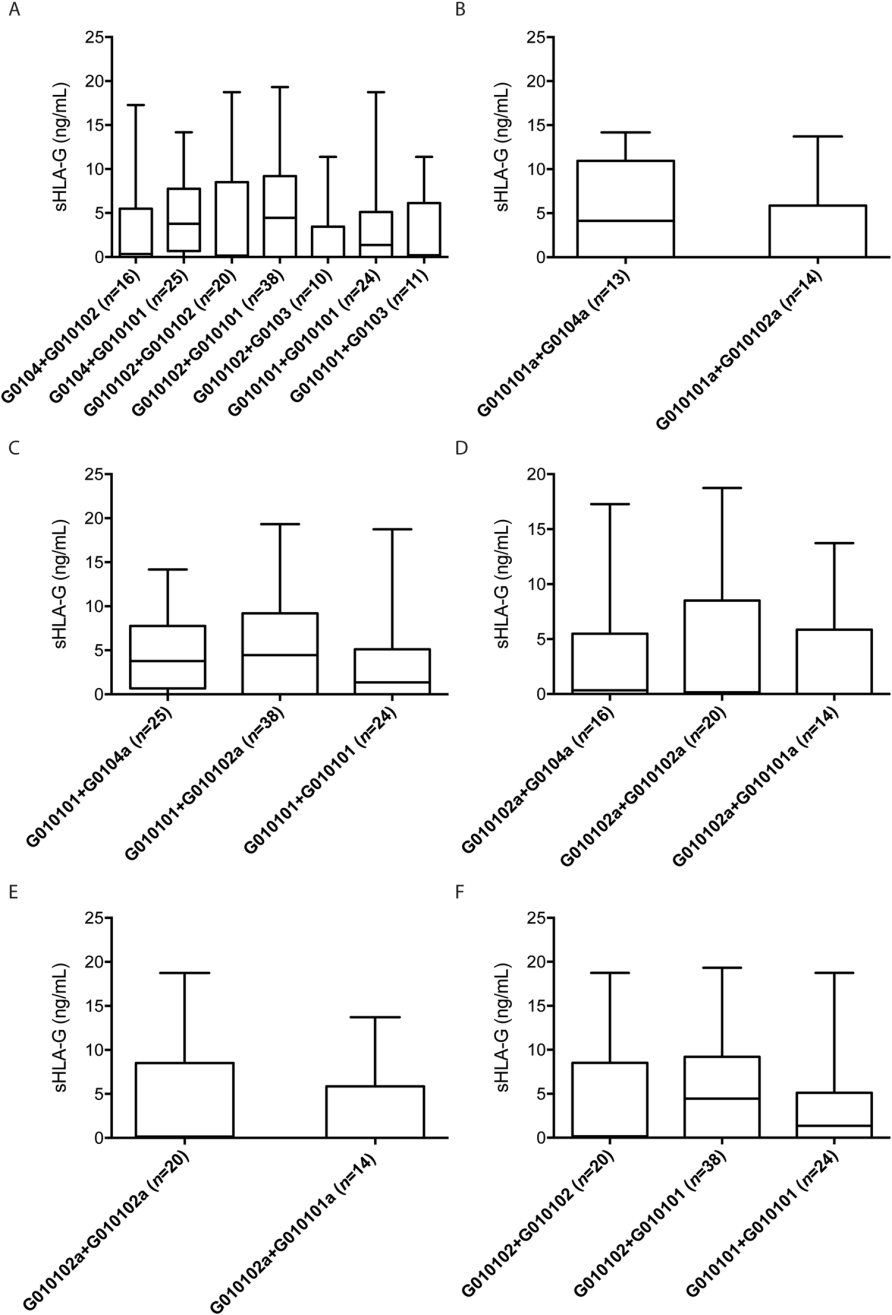
Association analysis between the soluble HLA-G (sHLA-G) levels and the *HLA-G* 5′URR diplotypes (pairs of haplotypes) in a Brazilian population sample (only those groups with at least ten occurrences were considered), stratified as follows: (**A**) the whole sample was stratified according to diplotypes of promoter lineages. The statistical analysis was performed using Kruskal-Wallis test followed by the Dunn’s posttest. (**B**) Diplotypes composed of at least one copy of the G010101a promoter, which is the second most frequent haplotype. The statistical analysis was performed using Wilcoxon matched pairs test. (**C**) Diplotypes composed of at least one copy of the G010101 promoter, which is the most frequent lineage. The statistical analysis was performed using Kruskal-Wallis test followed by the Dunn’s posttest. (**D**) Diplotypes composed of at least one copy of the G010102a promoter, which is the most frequent haplotype. The statistical analysis was performed using Kruskal-Wallis test followed by the Dunn’s posttest. (**E**) Diplotypes composed of the two more frequent promoter haplotypes: G010101a and G010102a. The statistical analysis was performed using Wilcoxon matched pairs test. (**F**) Diplotypes composed of the two more frequent promoter lineages: G010101 and G010102. The statistical analysis was performed using Kruskal-Wallis test followed by the Dunn’s posttest. The data represent the median with the minimum and maximum values. *P < 0.05.

The association between sHLA-G levels and *HLA-G* 5′URR variable sites, genotypes and haplotypes disclosed no significant differences (data not shown). Nonetheless, the analysis of diplotypes (pair of haplotypes) (Fig. 4) revealed that the most frequent haplotype (G010102a) was observed together with several other haplotypes. However, no association was observed between sHLA-G levels and the different diplotypes. For this analysis, we considered only diplotype groups with at least 10 occurrences.

## Discussion

HLA-G has been described to be a tolerogenic molecule and its neoexpression was associated with the modulation of several pathological conditions^40^. Therefore, the understanding of the factors that participate on the *HLA-G* expression regulation is clinically relevant. The regulatory *HLA-G* 5′URR and 3′UTR segments exert a crucial role on transcriptional and post-transcriptional gene regulation, respectively^13^. Variable sites identified along these segments influence or are suspected to modulate HLA-G expression. *HLA-G* 3′UTR variations have been shown to modulate mRNA stability^17–19^ through several potential mechanisms including the differential affinity of the *HLA-G mRNA* to microRNAs^22,41^. Variable sites along the *HLA-G* 5′URR were investigated mainly by population studies^20,27,29,30^ and were proposed to regulate the interaction of transcription factors with promoter binding sequences and DNA methylation^13^. Considering such hypothesis, we brought here new insights on the impact of the *HLA-G* 5′URR diversity on the gene transcription activity *in vitro* and on sHLA-G plasma levels in a cohort of healthy donors.

Firstly, we investigated the promoter activity level of ten frequently observed *HLA-G* 5′URR haplotypes using a luciferase reporter gene assay. All *HLA-G* 5′URR transfected haplotypes induced the luciferase activity in both HLA-G^+^ cell lines. Seven haplotypes provided higher response in FON^+^ than in JEG-3 cells, a result that is in line with the higher HLA-G expression observed in the melanoma cells^19,42^. This effect was not observed with three haplotypes, suggesting that the action of transcription factors that are qualitatively and/or quantitatively specific for each cell line was affected by the 5′URR polymorphisms. In addition, although differences in the levels of luciferase activities were observed between several haplotypes, few of them reached significance. For instance, nucleotide variations between haplotypes belonging to Promo-G0104 and Promo-G0103 lineages (16 SNP locations^20,27^) have no significant effect whatever the cell line used. On the contrary, the −541G and −483G alleles are exclusively found in G010101f and G010101d haplotypes, respectively, and are the only differences between them. Therefore, it is likely that these nucleotide variations are involved on the differential level of luciferase activity obtained with these haplotypes in JEG-3 cells. Noteworthy, the G010102a haplotype, which exhibited the lower activity in FON^+^ cells, strongly differs (13 specific variants) from haplotypes belonging to Promo-G010101 and Promo-G0103 lineages (Fig. 3). Considering the 16 variants with no apparent effect, −1140T allele is thus the only that might influence the low level of luciferase activity observed with the G010102a haplotype. Interestingly, the −1140 A/T SNP has been recently pointed out as a putative target for balancing selection, with -1140T allele hypothesized to be associated with a lower HLA-G expression than -1140A allele^27^. Otherwise, we found that the G010101a haplotype exhibited the highest activity when transfected into the JEG-3 cells, whereas the G010101b haplotype exhibited the highest activity when transfected into FON^+^ cells. Considering the activity of G010101a and G010101b haplotypes, the result obtained with FON^+^ cells is in agreement with the previous study performed by the Ober’s group using JEG-3 cells, reporting that the G010101b haplotype exhibited the highest activity compared to G010101a, G010101c, G010102 and G010301 haplotypes^24,28^. Noteworthy, the unique difference between G010101a (−725C) and G010101b (−725G) haplotypes is the polymorphic site observed at −725G/T/C, for which the −725G allele has been associated with increased expression levels^28^. However, the *HLA-G* 5′URR fragments used by Ober and colleagues (nucleotides from −1412 bp to −33 bp from ATG) were shorter than ours (−1438 bp to +2 bp). Therefore, one explanation for the apparent controversial results obtained with JEG-3 cells would be that specific factors involved in 5′URR responses could target the regions located −1438 bp to −1412 bp and/or −33 bp to + 2 bp and could interact with factors that might be associated with the −725G/T/C polymorphism. Interestingly, the Hviid’s group^43^ demonstrated no significant difference between the −725 C/C genotype and the −725 C/G genotype in the HLA-G cell surface expression of trophoblast cells from first trimester placental tissues. In agreement with the variations observed in luciferase activity with the −725G allele in the present study, the authors suggested that it could be due to high surface expression variation in the −725 C/G group.

With the aim to further explore the haplotype responses to known modulators of HLA-G expression, we observed that some SNPs were relevant to the level of luciferase activity. First, several lines of evidence indicate that IFN-β treatment up-regulates *HLA-G* expression and increases HLA-G levels^28,44,45^. Compared with untreated cells, we observed an increased luciferase activity with all 5′URR haplotypes transfected into JEG-3 cells cultured in the presence of IFN-β. This result was expected since the ten haplotypes contain an intact *HLA-G* ISRE. Among them, G010101b and G010101c are the only ones that exhibit the −725G variation site, and the luciferase activity of these haplotypes was significantly increased when compared to other haplotypes exhibiting the −725C or −725T alleles. As mentioned above, the role of this SNP has been studied by the Ober’s group using a site directed mutagenesis assay, corroborating the relevance of the −725G allele in the increased expression level. Nonetheless, and contrary to what was expected by testing less 5′URR haplotypes^28^, we found that the increased expression is also related to response to IFN-β. The SNP is located close to the *HLA-G* ISRE^28^ and thus might contribute to a specific DNA conformation and/or specific binding factor that would improve IRF-1 binding when the Guanine is present, a hypothesis to be investigated. Second, progesterone has an important role on the maintenance of pregnancy^33^ and increases HLA-G expression in JEG-3 cell line^46^. A functional binding site to progesterone receptor (PRE) has been reported located between the −52 bp and −38 bp^39^ at the *HLA-G* 5′URR and the closest variable site is located at −56C/T position. When JEG-3 transfectants were treated with progesterone, all except one haplotype (G010101a) increased the luciferase activity; however, only five *HLA-G* 5′URR haplotypes showed significant upregulation. Overall, these results are in agreement with those reported by Yie and colleagues (2006), who showed increased luciferase activity after progesterone treatment^39^. However, we observed that progesterone treatment had a limited effect on 5′URR activity compared to INF-β. Indeed, we can speculate that the progesterone response element in this region appears to be a contributing regulatory site to the *HLA-G* transcriptional level rather than a crucial one. Consequently, we cannot exclude the existence of additional PREs outside the 5′URR, which could increase *in vitro* the HLA-G expression^46^. Otherwise, Yie and colleagues did not evaluate the influence of the *HLA-G* 5′URR polymorphic sites on the HLA-G expression after progesterone treatment. Unexpectedly, our study revealed no relationship with any 5′URR variable site, including the 56 C > T SNP despite its proximity to PRE. Regarding this SNP, a lower HLA-G cell surface expression of trophoblast cells from first trimester was previously demonstrated with the -56 C/T genotype compared with the −56 C/C phenotype^43^. This SNP is located in a RREB1-binding site involved in HLA-G repression^37^ and thus might influence RREB1 binding rather than the progesterone response. Interestingly, regarding the most frequent worldwide haplotypes, the absence of progesterone effect is observed with the G010101a haplotype (2^nd^ most frequent), whereas a significant effect is observed with G010102a (1^st^ most frequent) and G0104a (3^rd^ most frequent) haplotypes^47^. Notably, the G010101a haplotype exhibits 13 variation sites compared with G010102a and G0104 haplotypes (Fig. 3)^20,27^; however, it differs only at the -541 position compared with G010101f, which exhibits a significant progesterone response. This suggests that a variable site or a specific combination of variable sites within G0104a, G010102a, G010101a, G010101f, G0103a and G0103e haplotypes may faintly up-regulate the luciferase activity observed upon progesterone treatment. Third, considering the influence of 5′URR polymorphism in the down regulation of the *HLA-G* expression, the steroidal alkaloid cyclopamine decreased the luciferase reporter gene expression for all transfected haplotypes, particularly for G010101a and G010101c. Cyclopamine acts as GLI (Glioma-Associated Oncogene)-1 and GLI-2 repressor or as GLI-3 inductor^48^. Whereas GLI-1 and GLI-2 act as transcriptional activators, GLI-3 functions as a transcriptional repressor^49–52^. The GLI-3 binding sequence identified between -1116 bp and −1108 bp is conserved in each haplotype and thus may firstly participate in the significant luciferase down-regulation observed following cyclopamine treatment. Otherwise, the binding sequence is close to the 1121 C > T variation site^38^ and the G010101c haplotype is the only one that presents a Thymine at position −1121 bp, whereas the others present a Cytosine. This suggests a possible influence of the −1121T variant on the magnitude of the cyclopamine response even if other SNPs are undoubtedly involved in the response level of the G010101a haplotype. Once again, and as proposed above for IFN-β, SNPs might participate in the DNA conformation and/or the binding of other factors that would modulate the action of cyclopamine according to the 5′URR haplotype.

Finally, regarding the possible association between plasma sHLA-G and *HLA-G* 5′URR polymorphisms we did not observe significant differences. However, significant associations have been previously identified with 3′UTR polymorphisms^23^, suggesting that sequence variations may primarily affect posttranscriptional mechanisms. Notably, we did not detect plasma sHLA-G in a large part of blood samples (44%; n = 157). Although the limit of sensitivity may differ according to the laboratory and the use of an in house or a commercial ELISA, this result is consistent with other studies^53,54^, one of which reported only 23% sHLA-G + plasmas (n = 30) from healthy donors. Interestingly and in agreement with a previous study^25^, we observed that the G010101b haplotype was underrepresented among sHLA-G^−^ donors. This haplotype was associated with increased HLA-G expression in FON^+^ cell line, and is in linkage disequilibrium with the 3′UTR haplotype known as UTR-4 and the coding G*01:01:01:05 allele^20,30^. Interestingly, UTR-4 has been classified as medium sHLA-G producers in previous studies^23,47^ and the G*01:01:01:05 allele presents different intronic sequences when compared to its counterparts the G*01:01:01 allele group, which may somehow influence alternative splicing. In addition, although luciferase activity provided by G010101b and G0103e haplotypes was similar in JEG-3 cells, we noticed a significant down-regulation when the G0103e haplotype was transfected into FON^+^ cells. In addition, the G0103e haplotype is mostly associated with UTR-5, previously associated with low sHLA-G production^19,23^.

In conclusion, all these findings are consistent with a moderate impact of the 1.4 kb 5′URR polymorphism on the magnitude of HLA-G expression in response to differential cellular microenvironment modulators. They also suggest that the 5′URR segment alone is not a high predictor of HLA-G expression level.

## Methods

### Cloning of the *HLA-G* 5′URR haplotypes into the pGL3-basic vector

DNA samples from seven Brazilian individuals carrying the ten main *HLA-G* 5′URR haplotypes (Table 1)^20,24,27,29,30^ were selected for the cloning procedure. DNA was amplified using the forward 5′-AAGCTTCACAAGAATGAGGTGGAGC and reverse 5′-CGCGGATCCTTGGCGTCTGG primers, generating a 1438 bp fragment. Cycling conditions consisted of 35 cycles of 30 s at 95 °C, 30 s at 60 °C and 1 min at 72 °C. PCR-amplified fragments were first inserted into pUCm-T vector (Bio Basic, Ontario, Canada) and confirmed by *Hind*III (Invitrogen, Carlsbad, CA) digestion. Ten constructions carrying each of the main haplotypes were selected and identified by Sanger’s sequencing analysis (Applied Biosystems, Foster City, CA). A 1525 bp *Kpn*I/*Bam*HI (Invitrogen) fragment obtained from each selected clone was subcloned into pGL3-Basic vector (Promega, Madison, WI) upstream of the firefly luciferase gene.

### *HLA-G* 5′URR sequence transfections and dual-luciferase report assay

Cells constitutively expressing HLA-G (choriocarcinoma JEG-3 and melanoma FON^+^ cell lines) were grown in DMEM (Gibco, Carlsbad, CA) and RPMI-1640 (Sigma-Aldrich, Lyon, France) supplemented mediums, respectively^55,56^. One microgram of each construction was transfected into sub-confluent JEG-3 cells by Lipofectamine 2000 Transfection Reagent (Invitrogen) and FON^+^ cells by Lipofectamine LTX Reagent with Plus Reagent (Invitrogen), according to manufacturer’s instructions. The pGL3-Promoter (Promega) and pGL3-Basic empty vectors were used as positive and negative controls, respectively. The pRL.SV40 Renilla Luciferase vector (Promega) was co-transfected to normalize the transfection efficiency.

Transfected cells were harvested and lysed 48 hours post-transfection. The supernatant was collected and used to perform the dual-luciferase report assay (Promega), according to manufacturer’s instructions. Firefly luciferase values were first normalized to those of Renilla luciferase values, and then to luciferase expression of the empty pGL3-Promoter vector. Assays were run in duplicates from at least four independent experiments.

Transfected JEG-3 cells were also treated for 24 hours with 5 µM cyclopamine (Tocris Bioscience, Minneapolis, MN), or 1000 U/mL interferon-β (RayBiotech, Norcross, GA) or 1 µg/mL progesterone (Sigma-Aldrich, France). At least four independent experiments were performed in duplicate. In parallel with treated cells, untreated transfected JEG-3 cell line was also cultured with medium (baseline), performing 12 independent experiments. Normalized Luciferase activities obtained with each *HLA-G* 5′URR constructions transfected into JEG-3 and FON^+^ cell lines and into JEG-3 cell line treated or not with interferon-β, progesterone or cyclopamine are presented in the Supplementary Tables S1 and S2.

### Subjects and DNA extraction

The study protocol was approved by the Ethics Committee of the Ribeirão Preto Medical School, University of São Paulo, Brazil (Protocol #6102/2013). A total of 157 unrelated healthy bone marrow donors (mean age = 32.31 ± 9.74) of both sexes (74 men) were randomly selected at the University Hospital of the same Institution. All methods were carried out in accordance with relevant guidelines and regulations. Written informed consent was obtained from all subjects. Genomic DNA was extracted from peripheral blood leucocytes using a standard salting out procedure^57^.

### *HLA-G* 5′URR typing

A 1752 bp fragment including the 5′URR segment was amplified and sequenced (using primers G-908R, G-830F, G-304R and GPR-247) as previously described^30^. The amplified fragment included 1446 nucleotides upstream the first translated ATG, and 388 nucleotides of the coding sequence. List of variable sites at *HLA-G* 5′URR region is presented in the Supplementary Table S3.

*HLA-G* 5′URR haplotypes were inferred using the PHASE algorithm^58^ as previously described^30^, and named according to previous studies^20,30,47^ since no official nomenclature has been assigned to this gene segment.

### Soluble HLA-G (sHLA-G) quantification

The sHLA-G plasma level was quantified in plasma by sandwich ELISA using mAb anti-HLA-G MEM-G/9 (Exbio, Praha, Czech Republic) and anti-β2-microglobulin (DAKO, Glostrup, Denmark) as capture and detection antibodies, respectively, as previously described^23,59^.

### Statistical analysis

Haplotype frequencies were estimated by direct counting, and adherence to the Hardy–Weinberg equilibrium (HWE) was tested using the GENEPOP 3.4 software^60^. Linkage disequilibrium (LD) between *HLA-G* SNPs was evaluated by means of Lewontin’s standardized coefficient *D’* and by a likelihood ratio test of LD implemented with the ARLEQUIN software^61^.

Promoter activity of the untreated transfectants was compared among all haplotypes using Kruskal-Wallis test followed by the Dunn’s posttest. For a given haplotype, the Wilcoxon matched pairs test was used for comparisons between treated (cyclopamine, interferon-β, or progesterone) and untreated transfectants. Comparisons between the effects (ratio or difference of expression) of a given treatment on the modulation of luciferase expression by different promoter haplotypes were performed either by the Kruskal-Wallis (followed by the Dunn’s posttest) or the Mann-Whitney tests. Associations among sHLA-G levels and 5′URR haplotypes and diplotypes (pair of haplotypes) were also performed by the Kruskal-Wallis or the Mann-Whitney tests. Only diplotypes groups with at least 10 occurrences were considered for association analysis with sHLA-G levels.

All these statistical analyses were performed using GraphPad Prism 5 v5.0b software.

## Electronic supplementary material

Dataset 1
