# Supplementary material for: The genetic diversity within the 1.4 kb HLA-G 5′ upstream regulatory region moderately impacts on cellular microenvironment responses
Source: Sci Rep. 2018 Apr 4;8:5652. doi: 10.1038/s41598-018-24009-7 (PMC5884815; doi:10.1038/s41598-018-24009-7)
Supplement: Supplementary file 1 — Dataset 1 [file 41598_2018_24009_MOESM1_ESM.doc]

**Supplementary Tables**

**The genetic diversity within the 1.4 kb *HLA-G* 5’ upstream regulatory region moderately impacts on cellular microenvironment responses.**

Fabrício C. Dias, Bruna C. Bertol, Isabelle Poras, Bruno M. Souto, Celso T. Mendes-Junior, Erick C. Castelli, Laure Gineau, Audrey Sabbagh, Nathalie Rouas-Freiss, Edgardo D. Carosella, Eduardo A. Donadi, Philippe Moreau

**Supplementary Table S1.** Normalized Luciferase activities obtained with each *HLA-G* 5’URR constructions transfected into JEG3 and FON^+^ cell lines, both HLA-G^+^.

|  | **Haplotypes** | | | | | | | | | | | | | |  |
| --- | --- | --- | --- | --- | --- | --- | --- | --- | --- | --- | --- | --- | --- | --- | --- |
| **Cell lines** | **G0104a** | **G0104b** | **G010102a** | **G010101a** | **G010101b** | **G010101c** | **G010101d** | | **G010101f** | | **G0103a** | | **G0103e** | |  |
| **JEG3** | 0.0285 | 0.0324 | 0.0278 | 0.0560 | 0.0358 | 0.0419 | | 0.0253 | | 0.0412 | | 0.0328 | | 0.0383 | |
|  | 0.0319 | 0.0337 | 0.0300 | 0.0565 | 0.0335 | 0.0373 | | 0.0243 | | 0.0408 | | 0.0311 | | 0.0351 | |
|  | (0.0101) | (0.0078) | (0.0098) | (0.0140) | (0.0095) | (0.0126) | | (0.0095) | | (0.0148) | | (0.0106) | | (0.0113) | |
|  | *n* = 24 | *n* = 24 | *n* = 24 | *n* = 24 | *n* = 24 | *n* = 24 | | *n* = 24 | | *n* = 24 | | *n* = 24 | | *n* = 24 | |
|  |  |  |  |  |  |  |  | |  | |  | |  | |  |
| **FON^+^** | 0.0442 | 0.0585 | 0.0232 | 0.0506 | 0.1202 | 0.0590 | 0.0566 | | 0.0512 | | 0.0658 | | 0.0465 | |  |
|  | 0.0467 | 0.0715 | 0.0279 | 0.0488 | 0.1251 | 0.0688 | 0.0596 | | 0.0526 | | 0.0662 | | 0.0478 | |  |
|  | (0.0115) | (0.0610) | (0.0098) | (0.0155) | (0.0427) | (0.0443) | (0.0176) | | (0.0149) | | (0.0231) | | (0.0133) | |  |
|  | *n* = 10 | *n* = 10 | *n* = 10 | *n* = 10 | *n* = 8 | *n* = 10 | *n* = 10 | | *n* = 10 | | *n* = 10 | | *n* = 10 | |  |

Median, Mean expression, Standard Deviation (SD) and *n* = Sample sizes are represented.

**Supplementary Table S2.** Normalized Luciferase activities obtained with each *HLA-G* 5’URR constructions transfected into JEG3 HLA-G^+^ cell line treated or not with interferon-β, progesterone or cyclopamine.

|  |  | **Haplotypes** | | | | | | | | | | | | | | |  |
| --- | --- | --- | --- | --- | --- | --- | --- | --- | --- | --- | --- | --- | --- | --- | --- | --- | --- |
| **Treatment** |  | **G0104a** | **G0104b** | **G010102a** | **G010101a** | **G010101b** | **G010101c** | | **G010101d** | | **G010101f** | | **G0103a** | | **G0103e** | |  |
| **Interferon-β** | - | 0.0242 | 0.0240 | 0.0226 | 0.0425 | 0.0227 | | 0.0219 | | 0.0148 | | 0.0295 | | 0.0208 | | 0.0231 | |
|  |  | 0.0241 | 0.0252 | 0.0221 | 0.0442 | 0.0242 | | 0.0217 | | 0.0136 | | 0.0261 | | 0.0191 | | 0.0214 | |
|  |  | (0.0019) | (0.0027) | (0.0020) | (0.0132) | (0.0074) | | (0.0051) | | (0.0034) | | (0.0083) | | (0.0041) | | (0.0051) | |
|  |  | *n* = 8 | *n* = 8 | *n* = 8 | *n* = 8 | *n* = 8 | | *n* = 8 | | *n* = 8 | | *n* = 8 | | *n* = 8 | | *n* = 8 | |
|  |  |  |  |  |  |  |  |  |  |  |  |  |  |  |  |  |  |
|  | + | 0.0470 | 0.0655 | 0.0469 | 0.0778 | 0.0835 | 0.0724 | | 0.0409 | | 0.0588 | | 0.0432 | | 0.0497 | |  |
|  |  | 0.0562 | 0.0631 | 0.0483 | 0.0949 | 0.0824 | 0.0691 | | 0.0414 | | 0.0572 | | 0.0458 | | 0.0548 | |  |
|  |  | (0.0266) | (0.0261) | (0.0178) | (0.0434) | (0.0351) | (0.0281) | | (0.0185) | | (0.0270) | | (0.0195) | | (0.0282) | |  |
|  |  | *n* = 8 | *n* = 8 | *n* = 8 | *n* = 8 | *n* = 8 | *n* = 8 | | *n* = 8 | | *n* = 8 | | *n* = 8 | | *n* = 8 | |  |
| **Progesterone** | - | 0.0332 | 0.0422 | 0.0307 | 0.0649 | 0.0424 | 0.0443 | | 0.0270 | | 0.0479 | | 0.0379 | | 0.0439 | |  |
|  |  | 0.0387 | 0.0417 | 0.0373 | 0.0636 | 0.0412 | 0.0475 | | 0.0274 | | 0.0471 | | 0.0365 | | 0.0444 | |  |
|  |  | (0.0114) | (0.0047) | (0.0127) | (0.0060) | (0.0049) | (0.0068) | | (0.0045) | | (0.0084) | | (0.0074) | | (0.0037) | |  |
|  |  | *n* = 8 | *n* = 8 | *n* = 8 | *n* = 8 | *n* = 8 | *n* = 8 | | *n* = 8 | | *n* = 8 | | *n* = 8 | | *n* = 8 | |  |
|  |  |  |  |  |  |  |  |  |  |  |  |  |  |  |  |  |  |
|  | + | 0.0450 | 0.0452 | 0.0418 | 0.0577 | 0.0447 | 0.0495 | | 0.0298 | | 0.0625 | | 0.0456 | | 0.0493 | |  |
|  |  | 0.0496 | 0.0442 | 0.0477 | 0.0511 | 0.0454 | 0.0561 | | 0.0304 | | 0.0623 | | 0.0516 | | 0.0550 | |  |
|  |  | (0.0143) | (0.0101) | (0.0178) | (0.0188) | (0.0042) | (0.0213) | | (0.0045) | | (0.0198) | | (0.0136) | | (0.0147) | |  |
|  |  | *n* = 8 | *n* = 8 | *n* = 8 | *n* = 8 | *n* = 8 | *n* = 8 | | *n* = 8 | | *n* = 8 | | *n* = 8 | | *n* = 8 | |  |
| **Cyclopamine** | - | 0.0240 | 0.0269 | 0.0235 | 0.0470 | 0.0249 | 0.0272 | | 0.0166 | | 0.0319 | | 0.0221 | | 0.0254 | |  |
|  |  | 0.0238 | 0.0271 | 0.0228 | 0.0473 | 0.0252 | 0.0312 | | 0.0179 | | 0.0343 | | 0.0256 | | 0.0283 | |  |
|  |  | (0.0018) | (0.0030) | (0.0022) | (0.0118) | (0.0078) | (0.0100) | | (0.0035) | | (0.0053) | | (0.0058) | | (0.0056) | |  |
|  |  | *n* = 10 | *n* = 10 | *n* = 10 | *n* = 10 | *n* = 10 | *n* = 10 | | *n* = 10 | | *n* = 10 | | *n* = 10 | | *n* = 10 | |  |
|  |  |  |  |  |  |  |  |  |  |  |  |  |  |  |  |  |  |
|  | + | 0.0179 | 0.0206 | 0.0161 | 0.0282 | 0.0183 | 0.0199 | | 0.0151 | | 0.0243 | | 0.0224 | | 0.0238 | |  |
|  |  | 0.0179 | 0.0192 | 0.0154 | 0.0297 | 0.0180 | 0.0196 | | 0.0144 | | 0.0235 | | 0.0216 | | 0.0218 | |  |
|  |  | (0.0030) | (0.0048) | (0.0054) | (0.0114) | (0.0043) | (0.0084) | | (0.0029) | | (0.0044) | | (0.0044) | | (0.0047) | |  |
|  |  | *n* = 10 | *n* = 10 | *n* = 10 | *n* = 10 | *n* = 10 | *n* = 10 | | *n* = 10 | | *n* = 10 | | *n* = 10 | | *n* = 10 | |  |

Median, Mean expression, Standard Deviation (SD) and *n* = Sample sizes are represented.

**Supplementary Table S3.** List of variable sites at *HLA-G* 5’URR region.

| **hg19 Chr6 position** | **SNPid** | **Reference allele** | **Alternative allele** | **IMGT/HLA related position** | **Reference allele frequency (2n=314)** |
| --- | --- | --- | --- | --- | --- |
| 29794317 | rs1736936 | G | A | -1305 | 0.494 |
| 29794443 | rs1736935 | A | G | -1179 | 0.389 |
| 29794467 | rs3823321 | G | A | -1155 | 0.822 |
| 29794482 | rs1736934 | A | T | -1140 | 0.669 |
| 29794484 | rs17875389 | A | G | -1138 | 0.898 |
| 29794501 | rs3115630 | T | C | -1121 | 0.057 |
| 29794658 | rs1632947 | G | A | -964 | 0.490 |
| 29794860 | rs1632946 | C | T | -762 | 0.293 |
| 29794897 | rs1233334 | G | C,T | -725 | 0.121 |
| 29794906 | rs2249863 | T | G | -716 | 0.490 |
| 29794933 | rs2735022 | A | G | -689 | 0.490 |
| 29794956 | rs35674592 | G | T | -666 | 0.490 |
| 29794976 | rs17875391 | A | G | -646 | 0.968 |
| 29794989 | rs1632944 | G | A | -633 | 0.490 |
| 29795076 | rs201221694 | A | AG | -546 | 0.930 |
| 29795081 | rs368205133 | GA | G | -541 | 0.946 |
| 29795113 | rs17875393 | C | G | -509 | 0.927 |
| 29795136 | rs1736933 | A | C | -486 | 0.490 |
| 29795139 | rs149890776 | A | G | -483 | 0.984 |
| 29795145 | rs1736932 | C | G | -477 | 0.389 |
| 29795179 | rs17875394 | G | A | -443 | 0.990 |
| 29795222 | rs17875395 | G | A | -400 | 0.904 |
| 29795231 | rs17875396 | G | A | -391 | 0.904 |
| 29795253 | rs1632943 | C | A | -369 | 0.389 |
| 29795421 | rs1233333 | G | A | -201 | 0.490 |
| 29795566 | rs17875397 | C | T | -56 | 0.898 |
